# A 12-Week Randomized Controlled Trial of Nutrition and Exercise Education with Dietary Supplementation for Sarcopenia Prevention in Korean Baby Boomers

**DOI:** 10.3390/nu17183008

**Published:** 2025-09-20

**Authors:** Eun-Hee Jang, Seungmin Lee

**Affiliations:** Department of Food and Nutrition, Sungshin Women’s University, Seoul 01133, Republic of Korea; j.ehee26@gmail.com

**Keywords:** sarcopenia, nutrition education, exercise program, dietary supplementation, randomized controlled trial, Korean baby boomers

## Abstract

**Background/Objectives**: Sarcopenia is a major public health concern, and early preventive strategies in midlife are increasingly emphasized. The goal of this study was to evaluate the effectiveness of a 12-week lifestyle intervention that included nutrition education, exercise education, and dietary supplementation in Korean baby boomers. **Methods**: This single-blind, randomized controlled trial included 42 Korean baby boomers born between 1955 and 1963. Participants were randomly assigned to one of three groups: (1) nutrition and exercise education plus dietary supplementation (DiExSp), (2) nutrition and exercise education only (DiEx), or (3) control. The intervention was delivered online, and the DiExSp group additionally received a daily supplement containing protein, vitamins, and minerals. Primary outcomes included dietary intake, body composition, physical function, and fasting blood biomarkers. Nonparametric tests and effect size calculations were performed using SAS version 9.4. **Results**: A total of 31 participants completed the study. The results showed significant increases in protein and micronutrient intake (*p* < 0.05), with large effect sizes in the DiExSp group. Handgrip strength showed improvement in both the DiExSp and DiEx groups (DiExSp: *p* = 0.027, *r* = 0.63; DiEx: *p* = 0.020, *r* = 0.73), while no changes were observed in other physical parameters. HbA1c levels showed a significant decrease in the DiExSp group compared to the control (*p* < 0.05, ε^2^ = 0.01). No significant differences were observed for other biomarkers. **Conclusions**: A 12-week intervention combining dietary supplementation with education resulted in improved dietary intake, muscle strength, and glycemic control. However, most effects were confined to within-group changes. Between-group differences were minimal, and effect sizes were small. These findings provide preliminary evidence that lifestyle intervention strategies may contribute to sarcopenia prevention in midlife adults.

## 1. Introduction

Aging of the global population is occurring at an unprecedented rate, driven by increasing life expectancy and declining birth rates [1]. Consequently, age-related health issues have become a major public health concern. Among these conditions, sarcopenia has been recognized as a key geriatric syndrome, characterized by the progressive loss of skeletal muscle mass, strength, and function. Without timely intervention, sarcopenia may result in a range of health problems, including mobility limitations, metabolic disease, and cardiovascular disease, and can place a significant burden on healthcare systems around the world [2,3].

Sarcopenia has traditionally been studied in adults aged 65 years and older; however, based on recent evidence, preventive strategies and early management should be initiated during midlife. According to one study, approximately 9% of middle-aged women have experienced a significant decline in lean body mass over a three-year period, which is associated with reduced physical function [4]. This finding supports the idea that early intervention and lifestyle modification across the lifespan can play an essential role in prevention of sarcopenia and promotion of physical performance in later life [5]. Korean baby boomers, defined as individuals born between 1955 and 1963, constitute approximately 15% of the total population and have recently entered old age (≥ 65 years) [6]. One study found that the generation that experienced fetal and early childhood malnutrition during the Korean War (1950–1953) reported a higher risk of metabolic disease compared to baby boomers [7]. However, other studies suggest that the risk of chronic disease such as hypertension and diabetes may be higher for baby boomers compared with previous generations [8,9]. This may be due to nutritional deficiencies in baby boomers early in life and exposure to a Westernized diet since then. This cohort includes a large population with the potential of having a significant impact on future healthcare needs. Therefore, application of practical strategies to prevent sarcopenia in baby boomers is critical to public health.

Lifestyle factors, including nutrition, exercise, and dietary supplementation, are believed to play important roles in the prevention and management of sarcopenia. Healthy diet pattern, such as a Mediterranean diet, and resistance training have been shown to be helpful in maintenance of muscle health [10,11,12,13]. In particular, a synergistic effect of a combination of nutrition and exercise programs on improving body composition and physical function in older adults with sarcopenia has been demonstrated [14]. An integrated approach combining nutrition and exercise is recommended in the major international guidelines for management of sarcopenia from the Asian Working Group for Sarcopenia (AWGS), the European Society for Clinical and Economic Aspects of Osteoporosis and Osteoarthritis (ESCEO), and the International Conference on Frailty and Sarcopenia Research (ICSFR) [15,16,17]. These guidelines emphasize appropriate protein intake, dietary supplements with amino acids, vitamins, and minerals, and resistance training as key components. Although many studies on sarcopenia have been conducted, most of them have included older adults. Generalizing their findings to younger cohorts, such as Korean baby boomers, who need a preventative approach, is difficult.

Therefore, the aim of this study was to evaluate the exploratory effects of a 12-week intervention involving a lifestyle program including nutrition and exercise education with dietary supplementation, focusing on its potential contribution to strategies related to sarcopenia prevention in Korean baby boomers. The findings of this study are of public health significance because the research was conducted among Korean baby boomers, a large aging population that is expected to place a significant burden on the healthcare system in the future, and may provide preliminary evidence to inform practical strategies related to sarcopenia prevention in relatively younger cohorts.

## 2. Materials and Methods

### 2.1. Study Design

This study is a 12-week, randomized controlled trial with a three-arm parallel design, conducted at a single center in Seoul between November 2022 and October 2023. The study was designed to evaluate the exploratory effects of nutrition education, exercise education, and dietary supplementation on sarcopenia-related outcomes in middle-aged and older adults. Participants were randomly assigned to three groups: (1) nutrition and exercise education plus dietary supplementation (DiExSp), (2) nutrition and exercise education only (DiEx), or (3) control.

### 2.2. Participants

**Figure 1:**
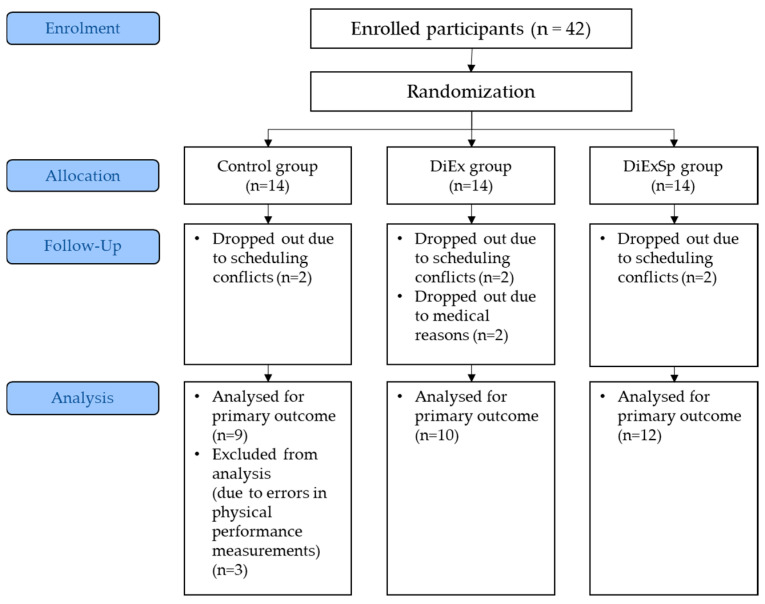
CONSORT 2025 flow diagram showing the progression of participants through each stage of the randomized controlled trial. DiEx, nutrition and exercise education only; DiExSp, nutrition and exercise education plus dietary supplementation.

A total of 42 participants were recruited through community outreach at public health centers, community centers, and universities located in Gangbuk-gu, Seoul, from May 2022 to July 2023. Eligibility screening was conducted in advance by trained graduate students by telephone or through face-to-face interviews to confirm inclusion and exclusion criteria, and all enrolled participants met the eligibility requirements. Participants were randomly assigned to one of three groups, and 31 participants completed the 12-week intervention and were included in the final analysis (see CONSORT flow diagram in Figure 1). Inclusion criteria are as follows: (1) healthy adults born between 1955 and 1963 who are part of the Korean baby boomer generation; (2) individuals capable of participating in nutrition and exercise programs; and (3) individuals with access to digital devices and the internet capable of taking online courses. The exclusion criteria are as follows: (1) engagement in regular resistance exercise, defined as participation at least once or twice per week over the past year (2) consumption of protein or androgen supplements within the past six months; (3) a diagnosis of diabetes or uncontrolled hypertension (defined as systolic blood pressure > 140 mmHg or diastolic blood pressure > 90 mmHg); (4) a history of kidney, cardiovascular, or pulmonary disease, or a cancer diagnosis within the past two years; (5) acute or chronic infectious diseases diagnosed by a physician.

### 2.3. Sample Size, Randomization, and Allocation

An a priori power analysis was performed using G*Power version 3.1.9.7 (Heinrich Heine University Düsseldorf, Germany) to determine the required sample size for a one-way analysis of variance (ANOVA). Assuming a large effect size (*f* = 0.56), a significance level of 0.05, and statistical power of 0.95, the estimated sample size was 54. To account for a potential 10% dropout rate, the final target sample size was set at 60 (20 per group). However, due to recruitment constraints during the Coronavirus Disease 2019 (COVID-19) pandemic, only 42 participants were enrolled. A revised power analysis using *f* = 0.50, α = 0.05, and power = 0.80 confirmed that the final sample size remained sufficient for detection of meaningful group differences.

All eligible participants were enrolled prior to randomization. After enrollment, participants were first listed and coded in the order of enrollment, and then assigned using a simple randomization procedure based on the random number generation algorithm in SAS version 9.4 (SAS Institute Inc., Cary, NC, USA). The randomization list was generated in a single batch by an independent researcher with no role in enrollment or intervention delivery. Randomization and group allocation were implemented only after completion of enrollment; assignment lists were inaccessible to study staff involved in enrollment or intervention, thereby preserving allocation concealment. To minimize potential bias, the control group also received general health information.

### 2.4. Interventions

Participants were randomly assigned to one of the following three groups: (1) DiExSp group; (2) DiEx group; and (3) a control group. Due to delays in participant recruitment, the intervention was conducted in two separate phases: the first from 28 November 2022 to 17 February 2023, and the second from 12 July 2023 to 2 October 2023. Each phase included 21 participants.

Both the DiExSp and DiEx groups received one baseline face-to-face nutrition education session, followed by weekly 15 to 30 min live remote nutrition education sessions and three 60 min live remote exercise sessions per week for 12 weeks. All sessions were conducted in real time using Zoom video conferencing software, version 5 (Zoom Video Communications, Inc., San Jose, CA, USA). Dietitians majoring in food and nutrition at Sungshin Women’s University provided nutrition education, and the exercise program was developed and supervised by professor and graduate students in physical education at the same institution.

The nutrition education component consisted of the following: (1) An introduction to the definition, causes, and associated risks of sarcopenia so that participants could recognize the importance of prevention. (2) Emphasis on the importance of adequate protein intake, including instruction on calculating individual requirements (1.5 g/kg body weight) based on findings from a randomized controlled trial in Koreans [18], and guidance on evenly distributing protein intake across three meals. (3) Emphasis on nutrients such as vitamin D, omega-3 fatty acids, with emphasis on food sources like milk and dairy products, which are known to support muscle health. (4) Encouragement to maintain a balanced diet and a healthy weight. (5) Practical application through the provision of personalized daily meal plans.

The exercise program consisted of a 10 min warm-up, 40 min of resistance and aerobic exercise, and a 10 min cool-down. The program was conducted in the form of live online training with supervision. The resistance exercises included arm curls, pull-downs, squats, and knee crunches. Aerobic exercises included walking and kicksteps. Exercise intensity was monitored by an exercise professional, the warm-up and cool-down phases were kept at a perceived exertion level of 10 to 12, and the main workout was performed at a perceived exertion level of 13 to 15 on the Borg Perceived Exertion Rating Scale.

Participants in the DiExSp group, in addition to receiving the same education as those in the DiEx group, received a powdered protein-based dietary supplement (Life Salad Wellcare Shake L2, Life Salad Co., Ltd., Goyang, Republic of Korea; manufactured by MLO Korea Co., Ltd., Paju, Republic of Korea) by mail and were instructed to consume 50 g dissolved in water once daily. Each serving provided 200 kcal, 24 g of protein, 5.5 g of fat, and 17.5 g of carbohydrates, as well as micronutrients. Details of the supplement’s nutritional composition and ingredients are provided in Supplementary Table S1.

The control group received printed and digital materials containing general health information, including nutrition and physical activity guidelines at baseline, and participated in monthly 15 to 30 min remote health education sessions. This group did not receive any structured exercise training or supplementation, thereby functioning as a minimal-intervention comparator. To isolate the effect of supplementation while balancing contact and educational exposure, the DiEx group served as an active comparator to the DiExSp group, enabling direct assessment of the incremental contribution of supplementation under otherwise similar intervention intensity.

No concomitant treatments or health programs beyond the assigned interventions were allowed during the trial. All participants were instructed to maintain their usual lifestyle, and no additional dietary supplements, structured exercise programs, or medications affecting muscle metabolism were permitted. Intervention (education and dietary supplements) was considered low risk, and participants were instructed to report any physical discomfort or abnormal symptoms during the intervention period, but no such cases were reported. Both the nutrition and exercise education components were completed by all participants. Individual make-up classes were provided for those who missed scheduled sessions to ensure full completion of the program. In particular, make-up sessions for the exercise education were conducted via Zoom, during which video recordings of the exercises were shared and participants’ performance was supervised in real time. This approach ensured 100% compliance with exercise sessions among participants who completed the study and maintained standardized procedures across all training. In the DiExSp group, weekly monitoring by a nutrition researcher confirmed that the prescribed dosage of the supplement was consistently consumed by all participants.

### 2.5. Outcome Measures and Data Collection

All outcome variables were assessed under the same conditions at baseline and immediately following the 12-week intervention. The primary outcome measures included dietary intake, body composition, physical function, and fasting blood biomarkers related to glucose metabolism, lipid metabolism, protein status, and micronutrient levels.

Dietary intake was assessed over three days using the 24 h dietary recall method. The recalls were conducted via real-time remote interviews by dietitians. An analysis of intakes of total energy, macronutrients (carbohydrates, protein, and fat), calcium, vitamin D, and other key nutrients was performed using CAN Pro 6.0 (The Korean Nutrition Society, Seoul, Republic of Korea).

Body height was measured using a digital stadiometer (GM-1000, Neogmtech, Seoul, Republic of Korea), and body composition was assessed using multi-frequency bioelectrical impedance analysis (InBody 770, InBody Co., Ltd., Seoul, Republic of Korea). Measurements were taken in the morning after an overnight fast; participants wore light clothing and no shoes. Parameters included body weight, fat-free mass, skeletal muscle mass, and body fat percentage.

Handgrip strength was measured using a digital handgrip dynamometer (TKK 5404, Takei Scientific Instruments Co., Ltd., Niigata, Japan); one trial was performed for each hand (right and left), and the average value was used for analysis. Physical function was evaluated using the Short Physical Performance Battery (SPPB), consisting of three components: the chair stand test, 4 m walking speed, and balance test [19]. For the chair stand test, participants were instructed to rise from a chair five times as quickly as possible, with arms crossed over the chest, without using their arms. The total time to complete five repetitions was recorded in seconds. Gait speed was assessed over a 4 m course at the participant’s usual walking pace. Two trials were conducted, and the faster of the two was used for analysis. The balance test required participants to hold side-by-side, semi-tandem, and tandem stances for 10 s each. Each component was scored on a scale from 0 to 4, with a total SPPB score ranging from 0 to 12, where higher scores indicate better lower extremity function. All physical function assessments and body composition measurements were performed by graduate students trained in physical education following standardized procedures.

Blood tests were performed at a hospital-based health screening center (Our Best Internal Medicine Clinic, Gangbuk-gu, Seoul, Republic of Korea) using venous blood samples collected after ≥12 h of fasting. An analysis of the following biomarkers was performed: total protein, albumin, total bilirubin, blood glucose, glycated hemoglobin (HbA1c), insulin, blood urea nitrogen (BUN), creatinine, total cholesterol, high-density lipoprotein (HDL) cholesterol, low-density lipoprotein (LDL) cholesterol, triglycerides, and vitamin D. All analyses were performed by an external clinical laboratory commissioned by the hospital as part of routine medical testing. While standardized diagnostic procedures were presumably applied, detailed information on the analytical instruments and assay methods was not available.

Baseline demographic and health-related characteristics, including age, sex, smoking status, alcohol consumption, physical activity, household income, education level, subjective health status, and the Sarcopenia Quality of Life were assessed using self-administered questionnaires. The Korean version of the Sarcopenia Quality of Life (SarQoL-K) questionnaire was used to evaluate sarcopenia-related quality of life, with a total score ≤ 52.4 indicating impaired quality of life associated with sarcopenia [20].

### 2.6. Statistical Analysis

A total of 42 participants were enrolled in the study, and 31 participants were included in the final analysis. Eight participants were excluded from all analyses because they dropped out and requested permanent deletion of their data, which made an intention-to-treat analysis infeasible. Therefore, the analyses were limited to participants with complete data. Three participants were excluded from analyses due to outliers on the physical function test. Values that exceeded ± 3 standard deviations (SD) from the mean were considered to be due to measurement error. These cases were excluded from all analyses for maintenance of consistency. Only participants with complete data for all outcomes were included in the analysis. Missing data were not imputed.

Due to the relatively small sample size and the non-normal distribution of the data nonparametric statistical methods were applied. Continuous variables were presented as means ± SD, and categorical variables were expressed as frequencies and percentages. Baseline characteristics were compared among the three groups using the Kruskal–Wallis test for continuous variables and Fisher’s exact test for categorical variables. Within group changes from baseline to post-intervention were assessed using the Wilcoxon signed-rank test, and between-group differences were analyzed using the Kruskal–Wallis test. In the case of a significant difference, post hoc pairwise comparisons were performed using the Wilcoxon rank-sum test with Bonferroni correction to adjust for multiple testing and reduce the risk of type I error. Effect sizes were calculated as *r* (|Z|/√n) for within-group comparisons and epsilon-squared (ε^2^ = H/[N^2^ − 1]) for between-group comparisons. Values of *r* were interpreted as small (0.10–<0.30), medium (0.30–<0.50), and large (≥0.50) [21]. Values of ε^2^ were interpreted as small (0.01–<0.06), medium (0.06–<0.14), and large (≥0.14) [22]. No interim analyses, early termination, or subgroup analyses were performed in this study.

All statistical analyses were performed using SAS software, version 9.4 (SAS Institute Inc., Cary, NC, USA).

### 2.7. Ethical Considerations

This study was approved by the Institutional Review Board (IRB) of Sungshin Women’s University (IRB No. SSWUIRB-2022-026; date of approval: 11 May 2022), and written informed consent was obtained from all participants prior to enrollment. The trial was registered retrospectively in the Clinical Research Information Service, a primary registry in the World Health Organization International Clinical Trials Registry Platform (ID: KCT0010757; date of registration: 15 July 2025; website: https://cris.nih.go.kr/cris/search/detailSearchEn.do?seq=30574, accessed on 15 July 2025). In addition, the study followed the CONSORT 2025 checklist for reporting randomized trials [23] (Supplementary File S1).

## 3. Results

### 3.1. Baseline Data of Participants

The baseline characteristics of the participants are shown in Table 1. A total of 31 participants were included in the analysis, with 9 participants in the control group, 10 participants in the DiEx group, and 12 participants in the DiExSp group. No statistically significant differences in any of the baseline variables were observed among the three groups. The mean age of participants was 63.89 ± 2.62 years in the control group, 63.20 ± 1.99 years in the DiEx group, and 63.58 ± 2.43 years in the DiExSp group, with no significant differences across the groups (*p* = 0.744). Most participants were female, and no statistically significant differences in the proportion of females were observed between the groups (*p* = 0.361). Most participants were non-smokers (*p* = 1.000), and similar alcohol consumption patterns were also observed across the three groups (*p* = 0.819). No statistically significant differences in physical activity levels, monthly average household income, or educational level were observed among the groups (*p* > 0.05). Most participants engaged in physical activity that was moderate to vigorous in intensity (600–3000 Metabolic Equivalent of Task), had an income level of upper-middle class or higher, and had completed high school or higher education. Subjective health status scores were 2.44 ± 0.73 in the control group, 2.70 ± 0.67 in the DiEx group, and 2.83 ± 0.72 in the DiExSp group, and no significant differences were observed among the three groups (*p* = 0.386). Sarcopenia-related quality of life scores were considered indicative of impaired quality of life when the total score was 52.4 points or below. In this study, the score for the control group was 75.61 ± 10.91, the score for the DiEx group was 70.84 ± 13.74, and the score for the DiExSp group was 73.16 ± 8.83, with no statistically significant differences observed among the groups (*p* = 0.799).

### 3.2. Changes in Dietary Intake

A summary of changes in energy and macronutrient intake following 12 weeks of intervention is shown in Table 2. In the DiExSp group, protein intake showed a significant increase (pre: 56.5 ± 11.5 g vs. post: 101.6 ± 24.4 g, *p* < 0.001), with a very large effect size (*r* = 0.88). Animal protein intake also showed a significant increase (pre: 29.9 ± 9.5 g vs. post: 42.6 ± 15.6 g, *p* = 0.034), with a large effect size (*r* = 0.61). Similarly, a significant increase in total fat intake was observed in the DiExSp group (pre: 37.1 ± 11.9 g vs. post: 52.5 ± 15.5 g, *p* = 0.021), with a large effect size (*r* = 0.66). In addition, intakes of several vitamins and minerals showed a significant increase in the DiExSp group (Supplementary Table S2). In the control and DiEx groups, no statistically significant changes were observed in most nutrient intake levels. Between-group comparisons revealed statistically significant differences in protein (*p* = 0.004, ε^2^ = 0.01), vitamin D (*p* < 0.001, ε^2^ = 0.02), vitamin E (*p* = 0.022, ε^2^ = 0.01), riboflavin (*p* = 0.006, ε^2^ = 0.01), and zinc (*p* = 0.018, ε^2^ = 0.01). However, all observed effect sizes were small (ε^2^ = 0.00–0.02).

**Figure 2:**
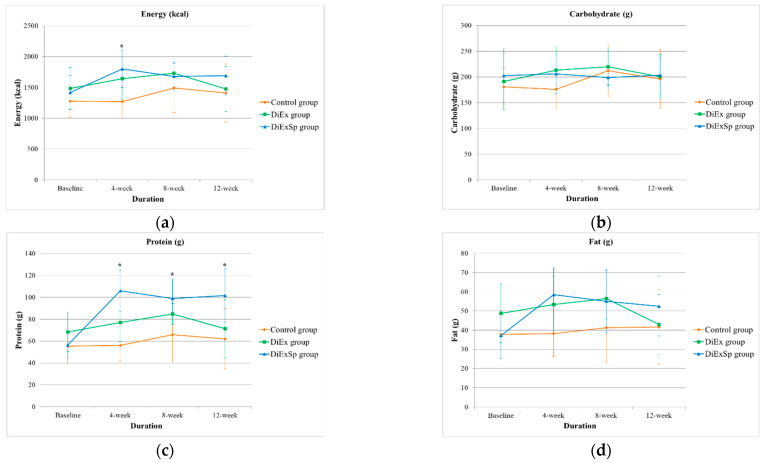
Changes in daily energy and macronutrient intake over the 12-week intervention period among the control, DiEx, and DiExSp groups. (**a**) Energy intake (kcal); (**b**) Carbohydrate intake (g); (**c**) Protein intake (g); (**d**) Fat intake (g). * Statistically significant differences between groups at the corresponding time point (*p* < 0.05 by Kruskal–Wallis test). DiEx, nutrition and exercise education only; DiExSp, nutrition and exercise education plus dietary supplementation.

The trends in energy and macronutrient intake over the 12-week intervention period are shown in Figure 2. Energy intake differed significantly among the three groups at the 4-week time point. However, no significant differences were observed at the 8-week and 12-week time points. Statistically significant differences in protein intake were observed among the groups at all three time points (*p* < 0.05). In contrast, no significant group differences in carbohydrate or fat intake were observed at any time point (*p* > 0.05). The results of post hoc comparisons with Bonferroni correction showed significantly higher protein intake in the DiExSp group compared to the control group at all measured time points from 4 to 12 weeks (Bonferroni-corrected *p* < 0.05). In addition, protein intake in the DiExSp group was significantly higher than that in the DiEx group at both the 4-week and 12-week time points (*p* < 0.05). No statistically significant differences in protein intake were observed between the DiEx group and the control group at any time point.

### 3.3. Changes in Body Composition and Physical Function

A summary of changes in body composition and muscle strength parameters following the 12-week intervention is shown in Table 3. In both the DiEx and DiExSp groups, handgrip strength showed a significant increase (DiEx, pre: 32.9 ± 5.9 kg vs. post: 35.7 ± 5.5 kg, *p* = 0.020, *r* = 0.73; DiExSp, pre: 39.5 ± 9.5 kg vs. post: 42.0 ± 8.2 kg, *p* = 0.027, *r* = 0.63), corresponding to very large and large effect sizes, respectively. No significant within-group changes were observed in other parameters, including body weight, body mass index (BMI), skeletal muscle mass, lean body mass, body fat percentage. In addition, no significant differences were observed between groups for all parameters (*p* > 0.05, ε^2^ = 0.00). There were no significant differences in physical function for SPPB between groups from baseline to week 12 (Supplementary Table S3).

### 3.4. Changes in Blood Biomakers

Changes in blood markers following 12 weeks of intervention are shown in Table 4. Among the analyzed blood markers, only HbA1c showed a statistically significant between-group difference (*p* = 0.043, ε^2^ = 0.01), and the effect size was interpreted as small. No statistically significant within-group changes were observed for any measured biomarker, including albumin, total bilirubin, fasting glucose, insulin, BUN, creatinine, total cholesterol, HDL cholesterol, LDL cholesterol, triglycerides, and vitamin D, in all three groups (*p* > 0.05). In addition, no statistically significant between-group differences in these variables were observed (*p* > 0.05, ε^2^ = 0.00).

**Figure 3:**
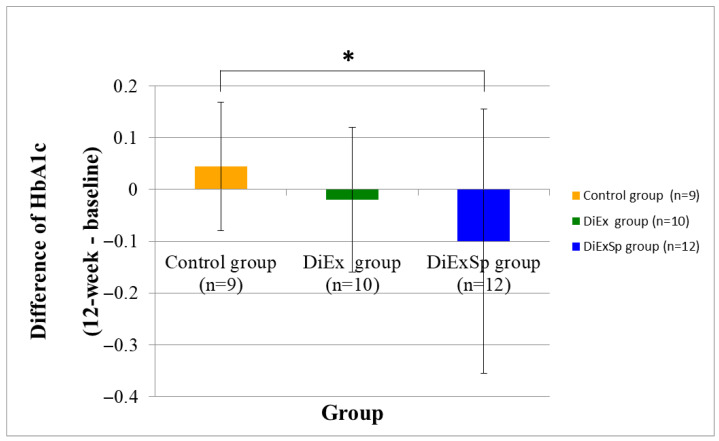
Comparison of changes in HbA1c levels (12-week minus baseline) among the control, DiEx, and DiExSp groups. * Statistically significant difference among groups (*p* < 0.05 by Bonferroni-adjusted Wilcoxon rank-sum test following Kruskal–Wallis analysis). DiEx, nutrition and exercise education only; DiExSp, nutrition and exercise education plus dietary supplementation.

As shown in Figure 3, Bonferroni-adjusted Wilcoxon signed-rank test revealed a significantly greater reduction in HbA1c levels in the DiExSp group compared to the control group (*p* < 0.05). However, no significant difference was observed between the DiExSp and DiEx groups (*p* > 0.05). The corresponding effect size (ε^2^ = 0.01) was interpreted as small.

## 4. Discussion

This randomized controlled trial evaluated the exploratory effects of a 12-week lifestyle intervention program combining nutrition and exercise education with a protein-based dietary supplement on sarcopenia-related outcomes in the Korean baby boomer generation. In the DiExSp group, which received nutrition and exercise education and supplementation, intakes of protein and key micronutrients significantly increased (large effect size), and handgrip strength also improved significantly (large effect size). Moreover, reductions in HbA1c levels were greater than in the control group, although the effect size was small. In the DiEx group, which received nutrition and exercise education alone, a significant improvement in handgrip strength was observed (large effect size), whereas no significant changes were found in nutrient intake or metabolic biomarkers. Overall, between-group differences were small and did not reach clinical significance, indicating that the comparative effects across groups were limited.

The significant increase in protein and intakes of several micronutrient only in the DiExSp group who received dietary supplements, as compared to the DiEx group who received the same nutrition education, is likely a result of supplementation. Recent international guidelines consistently recommend that older adults consume more protein–1.0 to 1.5 g/kg/day, depending on their health status [15,16,17,24]. Supplementation may therefore be necessary to achieve these targets, particularly in populations at risk of sarcopenia. The dietary supplement used in this study contained not only protein but also various micronutrients associated with muscle metabolism, such as vitamin D, calcium, and B vitamins. The combined effects of micronutrients may have supported the observed within-group improvements in strength. Vitamin D supports muscle protein synthesis and suppresses muscle atrophy [25], while minerals such as magnesium, selenium, and calcium have been implicated in sarcopenia prevention [26]. In addition, micronutrient-rich dietary patterns high in B vitamins, minerals, and antioxidants, have shown association with a reduced risk of sarcopenia [27].

Handgrip strength showed significant within-group improvement after the 12-week intervention in both the DiEx and DiExSp groups, which received the same nutrition and exercise education. The exercise program likely contributed meaningfully to strength gains, especially considering that no significant change in nutrient intake was observed in the DiEx group. Previous studies have also reported that a combination of aerobic and resistance training contributes to the maintenance of handgrip strength in middle-aged and older adults [28], and a meta-analysis reported that 1400 repetitions per week of resistance training was effective in improving handgrip strength in patients with sarcopenia [29]. Another study reported that neural adaptations occur more rapidly than morphological changes when older adults engage in resistance training [30], supporting the finding that improvements in strength may be the priority during the 12-week intervention. However, in our study these improvements were largely confined to within-group changes, while between-group differences were minimal and the effect sizes were small, indicating that the clinical significance of the observed strength gains remains limited and should be interpreted with caution.

The between-group change in HbA1c levels indicated a significantly greater reduction in HbA1c in the DiExSp group compared to the control group. These findings suggest that the addition of oral supplementation including protein and micronutrients to diet and exercise education may have contributed to improvements in glycemic regulation. Protein intake is associated with improved insulin sensitivity as well as maintenance of muscle mass [31,32], and micronutrients such as vitamins B and D, and calcium have also been reported to have a positive influence on insulin resistance [33,34,35]. Insulin resistance is a key mechanism in sarcopenia, impairing protein synthesis and promoting muscle catabolism [36]. Accordingly, the improvement in HbA1c observed in the current study may be a metabolically meaningful outcome that supports a physiological basis for sarcopenia prevention. However, the effect size was small and within-group changes were not significant, indicating that the clinical relevance of this result is limited and should be interpreted with caution as a preliminary finding.

No statistically significant changes in several major variables were observed in this study. First, no significant changes in intake of specific nutrients, including protein and micronutrients, were observed in the DiEx group that received nutrition and exercise education only. This is consistent with previous findings that many older adults are unable to meet protein requirements through diet alone [37], suggesting that education by itself may be insufficient to alter dietary patterns. Furthermore, no significant differences between groups were observed in other outcome measure except for HbA1c. A systematic review and meta-analysis reported that resistance exercise interventions longer than 12 weeks led to significant gains in muscle mass, whereas shorter interventions showed no effect [38], implying that the 12-week duration of our study may have been too short to detect meaningful changes. In addition, the subjects were a generally healthy cohort, with high levels of physical function and normal biochemical values, which may have produced a ceiling effect and reduced the likelihood of observing measurable intervention effects [39].

This study has several limitations. First, the relatively small sample size suggest that the findings should be interpreted with caution. We were unable to achieve the original target sample size due to recruitment challenges during the COVID-19 pandemic. However, a revised power analysis indicated that the statistical power remained sufficient. Nevertheless, the reduced sample size may have limited the statistical power to detect meaningful between-group differences, raising the possibility that true intervention effects were not observed. In addition, although non-parametric tests were applied and Bonferroni correction was used for post hoc pairwise comparisons, the potential for false-positive findings due to multiple testing cannot be entirely excluded. Second, participant dropout during the intervention period may have weakened the effect of randomization. Because dropouts requested deletion of their data, no intention-to-treat analysis could be performed, which may have introduced selection bias. Third, participants in the supplementation group were aware of their allocation, which may have introduced placebo or expectation bias. Fourth, differences in contact frequency between the control group and intervention groups may have introduced a Hawthorne effect, limiting the ability to isolate the effect of supplementation. To address this, we included an intervention group that received nutrition and exercise education without supplementation, which allowed us to better distinguish the effects of education from those of supplementation. Fifth, dietary intake assessed by 24 h recall may have recall bias and limited ability to capture day-to-day variability. Future studies should consider more frequent assessments or complementary methods. Sixth, the study population was limited to Korean baby boomers from a single urban area. Although the trial has value in addressing sarcopenia prevention in adults under 65 years and supporting nutrition–exercise strategies recommended by international guidelines, the generalizability of the findings is limited, highlighting the need for larger and more diverse studies. Finally, this study was registered retrospectively in the Clinical Research Information Service, which poses a limitation regarding research transparency and credibility. Nevertheless, it should be noted that all procedures were conducted under IRB approval and in accordance with the Declaration of Helsinki.

Despite these limitations, the findings of this study suggest that a combination of intervention strategies, including nutrition education, exercise education, and dietary supplementation, may contribute positively to the prevention of sarcopenia in Korean baby boomers. This relatively younger cohort is likely to increase future healthcare demands, emphasizing the public health importance of early preventive measures. Furthermore, integration of a digital, real-time platform for remote delivery of both nutrition and exercise components presents a feasible model for use in community-based interventions, particularly in the context of increasing demand for remote health strategies post COVID-19 pandemic. High levels of participation and adherence in this trial further support the internal validity of the findings. Future studies should examine the effects of the intervention in larger populations over a longer period of time and expand to a diverse population, including those at high risk for metabolic disease.

## 5. Conclusions

This randomized controlled trial evaluated the exploratory effects of a 12-week lifestyle intervention combining nutrition education, exercise education, and supplementation on the prevention of sarcopenia in Korean baby boomers. Significant improvements in nutrient intake and muscle strength were observed within the DiExSp group from baseline to post-intervention. However, no significant between-group differences were found. In contrast, HbA1c showed a significant decrease in the DiExSp group compared with the control group, although the effect size was small and no significant within-group changes were observed. No other outcome variables demonstrated significant differences. Overall, these findings suggest that the intervention produced some favorable effects, but the effect sizes were small and of limited clinical relevance. Future studies with larger sample sizes, longer intervention periods, and more diverse populations are warranted to confirm and extend these results.

## Figures and Tables

**Table 1:** Baseline characteristics of study participants.

Variables	Control Group (*n* = 9)	DiEx Group (*n* = 10)	DiExSp Group (*n* = 12)	*p* ^a^
**Age (yrs)**	63.89 ± 2.62	63.20 ± 1.99	63.58 ± 2.43	0.744
**Women (%)**	7 (77.8)	10 (100.0)	11 (91.7)	0.361
**Smoking status**				1.000
Non-smoker	8 (88.9)	9 (90.0)	10 (83.3)	
Former smoker	1 (11.1)	1 (10.0)	2 (16.7)	
Current smoker	0 (0.0)	0 (0.0)	0 (0.0)	
**Alcohol consumption status**				0.819
Non-drinker	5 (55.6)	4 (40.0)	6 (50.0)	
Drinker	4 (44.4)	6 (60.0)	6 (50.0)	
**Physical activity (METs-min/week)**				0.838
METs < 600	1 (11.1)	0 (0.0)	1 (8.3)	
600 ≤ METs < 3000	6 (66.7)	8 (80.0)	7 (58.3)	
3000 ≤ METs	2 (22.2)	2 (20.0)	4 (33.3)	
**Monthly household income**				0.722
Low (<1,000,000 won)	0 (0.0)	1 (10.0)	1 (8.3)	
Lower-middle (1,000,000–<2,000,000 won)	1 (11.1)	0 (0.0)	2 (16.7)	
Upper-middle (2,000,000–<4,000,000 won)	6 (66.7)	5 (50.0)	4 (33.3)	
High (≥4,000,000 won)	2 (22.2)	4 (40.0)	5 (41.7)	
**Education Levels**				0.273
Elementary school or below	0 (0.0)	0 (0.0)	0 (0.0)	
Middle school graduate	2 (22.2)	1 (10.0)	2 (16.7)	
High school graduate	3 (33.3)	3 (30.0)	8 (66.7)	
College graduate or above	4 (44.5)	6 (60.0)	2 (16.7)	
**Subjective health status**	2.44 ± 0.73	2.70 ± 0.67	2.83 ± 0.72	0.386
**Sarcopenia-specific Quality of Life score ^b^**	75.61 ± 10.91	70.84 ± 13.74	73.16 ± 8.83	0.799

Values are presented as mean ± SD or number (%). DiEx, nutrition and exercise education only; DiExSp, nutrition and exercise education plus dietary supplementation; METs, Metabolic Equivalent of Task; SD, standard deviation. ^a^ Fisher’s exact test was used for comparison of categorical variables, and the Kruskal–Wallis test was used for comparison of continuous variables. ^b^ A total score of ≤ 52.4 indicates a possible sarcopenia-related impairment in quality of life.

**Table 2:** Changes in energy and macronutrient intake (including dietary fiber and sugars) from baseline to 12 weeks within and between groups.

Variables	Control Group (*n* = 9)		*p* ^a^	*r* ^b^	DiEx Group (*n* = 10)		*p* ^a^	*r* ^b^	DiExSp Group (*n* = 12)		*p* ^a^	*r* ^b^	*p* ^c^	ε^2 d^
	Baseline	12-Week			Baseline	12-Week			Baseline	12-Week				
Energy (kcal)	1275.3 ± 256.4	1411.5 ± 470.5	0.570	0.22	1483.7 ± 335.3	1473.8 ± 361.7	0.922	0.05	1416.9 ± 275.9	1691.8 ± 317.6	0.077	0.52	0.309	0.00
Carbohydrate (g)	181.0 ± 37.4	196.2 ± 57.8	0.652	0.18	191.2 ± 54.7	200.5 ± 43.0	0.695	0.15	202.9 ± 51.6	203.5 ± 35.5	0.970	0.02	0.861	0.00
Fat (g)	37.8 ± 12.4	41.6 ± 19.4	0.426	0.30	48.7 ± 15.3	42.9 ± 15.6	0.432	0.27	37.1 ± 11.9	52.5 ± 15.5	**0.021**	0.66	0.110	0.00
Protein (g)	55.3 ± 15.8	62.0 ± 27.6	0.734	0.14	68.4 ± 17.9	71.3 ± 26.6	0.922	0.05	56.5 ± 11.5	101.6 ± 24.4	**<0.001**	0.88	**0.004**	0.01
Plant protein (g)	25.8 ± 6.1	28.1 ± 11.5	1.000	0.02	27.7 ± 11.1	30.9 ± 8.7	0.557	0.21	25.1 ± 5.9	31.6 ± 11.5	0.176	0.41	0.607	0.00
Animal protein (g)	26.1 ± 9.5	33.0 ± 20.6	0.301	0.38	37.4 ± 14.3	39.9 ± 23.0	0.770	0.11	29.9 ± 9.5	42.6 ± 15.6	**0.034**	0.61	0.199	0.00
Total dietary fiber (g)	24.7 ± 6.5	21.0 ± 7.1	0.250	0.41	25.0 ± 9.3	22.7 ± 6.0	0.625	0.18	20.9 ± 7.2	28.2 ± 8.7	0.110	0.48	0.079	0.01
Total sugars (g)	38.6 ± 16.8	29.8 ± 13.5	0.301	0.38	50.9 ± 20.2	36.2 ± 17.5	0.064	0.60	40.7 ± 21.6	32.9 ± 12.9	0.301	0.32	0.816	0.00

Values are presented as mean ± SD. DiEx, nutrition and exercise education only; DiExSp, nutrition and exercise education plus dietary supplementation; SD, standard deviation. ^a^ *p* values were obtained using the Wilcoxon signed-rank test for within-group comparisons between baseline and 12 weeks. ^b^ Effect sizes (*r*) corresponding to the Wilcoxon signed-rank test were calculated as |Z|/√n; values of *r* were interpreted as small (0.10–<0.30), medium (0.30–<0.50), and large (≥0.50). ^c^ *p* values were obtained using the Kruskal–Wallis test for comparison of changes from baseline to 12 weeks among the three groups. ^d^ Effect sizes (ε^2^) corresponding to the Kruskal–Wallis test were calculated as H/(N^2^ − 1), where H is the test statistic and N is the total sample size; values of ε^2^ were interpreted as small (0.01–<0.06), medium (0.06–<0.14), and large (≥0.14). Boldface indicates statistical significance (*p* < 0.05).

**Table 3:** Changes in body composition and muscle strength from baseline to 12 weeks within and between groups.

Variables	Control Group (*n* = 9)		*p* ^a^	*r* ^b^	DiEx Group (*n* = 10)		*p* ^a^	*r* ^b^	DiExSp Group (*n* = 12)		*p* ^a^	*r* ^b^	*p* ^c^	ε^2 d^
	Baseline	12-Week			Baseline	12-Week			Baseline	12-Week				
Height (cm)	158.3 ± 8.7	158.3 ± 8.7	-	-	155.7 ± 4.5	155.9 ± 4.3	-	-	156.0 ± 6.5	156.0 ± 6.5	-	-	0.350	0.00
Weight (kg)	58.3 ± 10.3	58.1 ± 9.7	0.844	0.10	56.8 ± 6.8	57.0 ± 6.6	0.449	0.24	59.5 ± 8.5	59.5 ± 8.0	0.952	0.02	0.893	0.00
BMI (kg/m^2^)	23.2 ± 3.0	22.8 ± 2.5	0.477	0.27	23.4 ± 2.3	23.4 ± 2.1	0.820	0.12	24.4 ± 3.1	24.7 ± 2.8	0.339	0.26	0.705	0.00
Skeletal muscle mass (g)	21.1 ± 4.2	21.6 ± 3.9	0.207	0.45	20.3 ± 2.4	20.4 ± 2.4	0.750	0.10	21.3 ± 2.6	21.1 ± 2.7	0.505	0.20	0.286	0.00
Lean body mass (g)	18.9 ± 4.2	18.1 ± 3.8	0.516	0.26	18.9 ± 4.9	19.0 ± 4.0	0.846	0.08	19.9 ± 6.1	20.3 ± 5.5	0.505	0.20	0.700	0.00
Body fat percentage (%)	32.3 ± 4.5	31.1 ± 3.7	0.426	0.30	33.0 ± 5.9	33.2 ± 4.5	0.770	0.11	33.0 ± 5.9	33.7 ± 5.5	0.392	0.24	0.487	0.00
Handgrip strength	39.8 ± 8.9	41.9 ± 10.4	0.129	0.53	32.9 ± 5.9	35.7 ± 5.5	**0.020**	0.73	39.5 ± 9.5	42.0 ± 8.2	**0.027**	0.63	0.724	0.00

Values are presented as mean ± SD. DiEx, nutrition and exercise education only; DiExSp, nutrition and exercise education plus dietary supplementation; BMI, Body mass index; SD, standard deviation. ^a^ *p* values were obtained using the Wilcoxon signed-rank test for within-group comparisons between baseline and 12 weeks. ^b^ Effect sizes (*r*) corresponding to the Wilcoxon signed-rank test were calculated as |Z|/√n; values of *r* were interpreted as small (0.10–<0.30), medium (0.30–<0.50), and large (≥0.50). ^c^ *p* values were obtained using the Kruskal–Wallis test for comparison of changes from baseline to 12 weeks among the three groups. ^d^ Effect sizes (ε^2^) corresponding to the Kruskal–Wallis test were calculated as H/(N^2^ − 1), where H is the test statistic and N is the total sample size; values of ε^2^ were interpreted as small (0.01–<0.06), medium (0.06–<0.14), and large (≥0.14). Boldface indicates statistical significance (*p* < 0.05).

**Table 4:** Changes in blood biomarkers from baseline to 12 weeks within and between groups.

Variables	Control Group (*n* = 9)		*p* ^a^	*r* ^b^	DiEx Group (*n* = 10)		*p* ^a^	*r* ^b^	DiExSp Group (*n* = 12)		*p* ^a^	*r* ^b^	*p* ^c^	ε^2 d^
	Baseline	12-Week			Baseline	12-Week			**Baseline**	**12-Week**				
Total protein (g/dL)	7.4 ± 0.4	7.5 ± 0.3	0.281	0.45	7.3 ± 0.4	7.4 ± 0.3	1.000	0.12	7.2 ± 0.5	7.2 ± 0.4	1.000	0.00	0.654	0.00
Albumin (g/dL)	4.6 ± 0.2	4.7 ± 0.2	0.328	0.38	4.6 ± 0.2	4.6 ± 0.2	1.000	0.00	4.6 ± 0.3	4.6 ± 0.3	0.656	0.20	0.637	0.00
Total bilirubin (mg/dL)	0.4 ± 0.1	0.4 ± 0.1	0.688	0.24	0.8 ± 0.4	0.7 ± 0.3	0.266	0.32	0.5 ± 0.2	0.6 ± 0.2	0.457	0.28	0.354	0.00
Blood glucose (mg/dL)	89.6 ± 5.3	90.9 ± 5.4	0.516	0.27	99.9 ± 10.6	98.9 ± 8.6	0.879	0.06	95.9 ± 8.4	94.6 ± 10.1	0.713	0.11	0.723	0.00
HbA1c (%)	5.8 ± 0.3	5.8 ± 0.2	0.438	0.54	6.1 ± 0.3	6.1 ± 0.3	0.275	0.32	6.1 ± 0.3	6.0 ± 0.4	0.233	0.55	**0.043**	0.01
Insulin (μIU/mL)	6.4 ± 4.6	5.7 ± 2.3	0.922	0.05	7.0 ± 4.3	7.5 ± 2.8	0.406	0.37	9.1 ± 7.0	7.0 ± 4.4	0.065	0.36	0.176	0.00
BUN (mg/dL)	16.6 ± 4.4	16.6 ± 3.0	1.000	0.04	15.9 ± 3.0	16.4 ± 3.6	0.766	0.11	16.3 ± 2.9	16.2 ± 3.4	0.957	0.02	0.964	0.00
Creatinine (mg/dL)	0.8 ± 0.1	0.7 ± 0.1	0.586	0.20	0.7 ± 0.1	0.7 ± 0.1	0.914	0.02	0.7 ± 0.2	0.7 ± 0.1	0.241	0.38	0.725	0.00
Total cholesterol (mg/dL)	193.1 ± 47.3	200.3 ± 34.5	0.453	0.28	181.8 ± 35.5	190.1 ± 31.9	0.369	0.31	183.0 ± 38.9	183.3 ± 38.8	0.985	0.01	0.582	0.00
HDL cholesterol (mg/dL)	62.9 ± 12.2	70.7 ± 17.8	0.055	0.65	67.1 ± 11.5	67.4 ± 13.7	1.000	0.00	58.3 ± 7.1	60.2 ± 9.5	0.551	0.22	0.163	0.00
LDL cholesterol (mg/dL)	112.8 ± 45.5	111.6 ± 32.8	0.992	0.02	94.0 ± 24.5	102.8 ± 27.0	0.264	0.37	101.5 ± 41.0	100.7 ± 39.1	0.979	0.01	0.347	0.00
Triglycerides (mg/dL)	87.1 ± 27.5	90.0 ± 34.9	0.934	0.04	104.2 ± 42.5	100.8 ± 27.4	0.752	0.11	116.1 ± 39.1	112.4 ± 26.9	0.953	0.02	0.970	0.00
Vitamin D (ng/mL)	37.7 ± 19.6	38.1 ± 22.6	0.590	0.18	45.7 ± 18.8	40.7 ± 17.5	0.125	0.50	28.2 ± 10.4	29.1 ± 10.8	0.677	0.14	0.179	0.00

Values are presented as mean ± SD. DiEx, nutrition and exercise education only; DiExSp, nutrition and exercise education plus dietary supplementation; HbA1c, glycated hemoglobin; BUN, blood urea nitrogen; HDL, high-density lipoprotein; LDL, low-density lipoprotein; SD, standard deviation. ^a^ *p* values were obtained using the Wilcoxon signed-rank test for within-group comparisons between baseline and 12 weeks. ^b^ Effect sizes (*r*) corresponding to the Wilcoxon signed-rank test were calculated as |Z|/√n; values of *r* were interpreted as small (0.10–<0.30), medium (0.30–<0.50), and large (≥0.50). ^c^ *p* values were obtained using the Kruskal–Wallis test for comparison of changes from baseline to 12 weeks among the three groups. ^d^ Effect sizes (ε^2^) corresponding to the Kruskal–Wallis test were calculated as H/(N^2^ − 1), where H is the test statistic and N is the total sample size; values of ε^2^ were interpreted as small (0.01–<0.06), medium (0.06–<0.14), and large (≥0.14). Boldface indicates statistical significance (*p* < 0.05).

## Data Availability

The data are not publicly available due to no explicit consent for data sharing was obtained from participants.

## Supplementary Materials

The following supporting information can be downloaded at: https://www.mdpi.com/article/10.3390/nu17183008/s1, Table S1. Nutritional Composition and ingredients of the dietary supplement (Life Salad Wellcare Shake L2, Life Salad Co., Ltd., Goyang, Republic of Korea; manufactured by MLO Korea Co., Ltd., Paju, Republic of Korea); Table S2. Changes in micronutrient intake from baseline to 12 weeks within and between groups; Table S3. Changes in physical function from baseline to 12 weeks within and between groups; File S1: CONSORT 2025 checklist of information to include when reporting a randomized trial.

## Abbreviations

The following abbreviations are used in this manuscript:

ANOVA	Analysis of variance
AWGS	Asian Working Group for Sarcopenia
BMI	Body mass index
BUN	Blood urea nitrogen
COVID-19	Coronavirus Disease 2019
DiEx	Nutrition and exercise education only
DiExSp	Nutrition and exercise education plus dietary supplementation
ESCEO	European Society for Clinical and Economic Aspects of Osteoporosis and Osteoarthritis
HbA1c	Glycated hemoglobin
HDL	High-density lipoprotein
ICSFR	International Conference on Frailty and Sarcopenia Research
IRB	Institutional Review Board
LDL	Low-density lipoprotein
METs	Metabolic Equivalent of Task
MUFA	Monounsaturated fatty acids
PUFA	Polyunsaturated fatty acids
RCTs	Randomized controlled trials
SarQoL-K	Korean version of the Sarcopenia Quality of Life
SD	Standard deviation
SFA	Saturated fatty acids
SPPB	Short Physical Performance Battery
