# Supplementary material for: A 12-Week Randomized Controlled Trial of Nutrition and Exercise Education with Dietary Supplementation for Sarcopenia Prevention in Korean Baby Boomers
Source: Nutrients. 2025 Sep 20;17(18):3008. doi: 10.3390/nu17183008 (PMC12472921; doi:10.3390/nu17183008)
Supplement: Supplementary file 1 [file nutrients-17-03008-s001.zip › nutrients-3854828-tables.pdf]

Table S1. Nutritional Composition and ingredients of the dietary supplement (Life Salad Wellcare Shake L2, Life Salad Co., Ltd., Goyang, Republic of Korea; manufactured by MLO Korea Co., Ltd., Paju, Republic of Korea).

Nutrient	Amount per 50 g
Energy (kcal)	200.00
Carbohydrate (g)	17.50
Sugars (g)	1.00
Protein (g)	24.00
Fat (g)	5.50
Saturated fatty acids (g)	2.00
Trans fatty acids (g)	< 0.25
Cholesterol (mg)	5.00
Sodium (mg)	60.00
Calcium (mg)	140.59
Vitamin D (μg)	10.00
Vitamin B_6 (mg)	0.30
Vitamin C (mg)	20.24
Vitamin E (mg α-TE)	2.02
Vitamin A (μg RAE)	151.63
Vitamin B_1 (mg)	0.24
Vitamin B_2 (mg)	0.30
Niacin (mg NE)	2.64
Folic Acid (μg)	81.00
Zinc (mg)	2.02
Iron (mg)	2.02
**Ingredients**	Whey protein isolate (Ireland), 15-grain mixed powder [rice (Republic of Korea), soybean (USA, China, Canada), whole wheat (USA), corn, glutinous rice, brown rice, sorghum, foxtail millet, black soybean, millet, black rice, adlay, peanut, sesame, carrot], medium-chain triglyceride (MCT) oil powder [MCT oil (Malaysia), glucose syrup, sodium caseinate, glycerin fatty acid esters, tricalcium phosphate], sunflower oil powder (imported), powdered cream [processed butter (Australia), lactose (USA), milk cream, whey protein, dipotassium phosphate, lecithin], 19-strain mixed probiotic powder, yam powder (Republic of Korea), beet powder (Republic of Korea), roasted salt (Republic of Korea), vitamin A powder, vitamin E powder, vitamin C, pyridoxine hydrochloride (vitamin B6), thiamine nitrate (vitamin B1), riboflavin (vitamin B2), folic acid, niacin, vitamin D, dicalcium phosphate, ferrous fumarate, zinc oxide.

Table S2. Changes in micronutrient intake from baseline to 12 weeks within and between groups.

Variables	Control group (n = 9)		p [a]	r [b]	DiEx group (n = 10)		p [a]	r [b]	DiExSp group (n = 12)		p [a]	r [b]	p [c]	ε^2 [d]
	Baseline	12-week			Baseline	12-week			Baseline	12-week				
Vitamin A (RAE) (μg RAE)	364.6 ± 165.5	370.7 ± 212.5	0.910	0.06	454.6 ± 327.4	387.5 ± 176.8	0.922	0.05	343.7 ± 85.9	487.7 ± 109.7	**0.001**	0.84	0.126	0.00

Retinol (μg)	103.9 ± 42.7	133.8 ± 124.1	0.426	0.30	186.3 ± 245.8	132.8 ± 64.0	0.846	0.08	107.8 ± 54.1	157.6 ± 83.1	0.204	0.38	0.763	0.00
Beta-carotene (μg)	3133.4 ± 1825.1	2875.7 ± 1488.9	0.496	0.26	3267.2 ± 2350.7	3083.8 ± 1552.2	0.492	0.24	2830.9 ± 941.7	2228.2 ± 914.4	0.064	0.54	0.129	0.00
Vitamin D (μg)	2.2 ± 1.3	1.6 ± 1.5	0.426	0.30	2.5 ± 1.9	2.9 ± 1.6	0.922	0.05	1.3 ± 1.1	12.4 ± 3.4	**<0.001**	0.88	**<0.001**	0.02
Vitamin E (mg)	11.8 ± 5.0	12.3 ± 6.4	1.000	0.02	11.1 ± 4.5	11.0 ± 3.8	1.000	0.02	9.0 ± 3.2	19.0 ± 10.0	**0.005**	0.77	**0.022**	0.01
Vitamin K (μg)	231.4 ± 165.3	254.6 ± 124.2	1.000	0.02	182.4 ± 86.1	238.5 ± 135.2	0.275	0.37	190.0 ± 81.7	185.4 ± 128.5	0.850	0.07	0.446	0.00
Vitamin C (mg)	60.5 ± 20.0	44.4 ± 24.4	0.129	0.53	60.9 ± 29.7	81.1 ± 47.3	0.625	0.18	57.6 ± 31.5	71.5 ± 28.8	0.233	0.36	0.146	0.00
Thiamin (mg)	0.9 ± 0.1	1.0 ± 0.3	0.203	0.45	0.9 ± 0.2	1.0 ± 0.3	0.492	0.24	0.9 ± 0.2	1.3 ± 0.3	**0.002**	0.82	0.073	0.01
Riboflavin (mg)	1.1 ± 0.2	1.2 ± 0.5	0.203	0.45	1.4 ± 0.3	1.4 ± 0.4	0.846	0.08	1.1 ± 0.3	1.9 ± 0.6	**0.001**	0.86	**0.006**	0.01
Niacin (mg)	7.8 ± 2.6	11.3 ± 7.6	0.203	0.45	9.9 ± 2.9	12.4 ± 5.8	0.275	0.37	9.4 ± 2.3	14.2 ± 4.2	**0.009**	0.72	0.310	0.00
Vitamin B_6 (mg)	0.3 ± 0.1	0.4 ± 0.2	0.820	0.10	0.3 ± 0.2	0.4 ± 0.3	0.275	0.37	0.5 ± 0.3	9.2 ± 29.5	0.110	0.48	0.508	0.00
Folate (DFE) (μg)	241.0 ± 83.8	261.6 ± 115.8	0.652	0.18	266.6 ± 84.6	268.3 ± 90.8	0.922	0.05	239.3 ± 70.0	352.2 ± 96.9	**0.016**	0.68	0.106	0.00
Vitamin B_{12} (μg)	3.1 ± 1.5	3.7 ± 5.7	0.570	0.22	3.1 ± 1.4	4.6 ± 5.9	0.922	0.05	1.7 ± 1.4	3.9 ± 2.6	**0.016**	0.68	0.105	0.00
Calcium (mg)	478.8 ± 175.6	472.2 ± 252.4	1.000	0.02	623.3 ± 325.9	620.6 ± 213.4	0.846	0.08	456.4 ± 140.5	766.7 ± 280.4	**0.003**	0.79	0.077	0.01
Phosphorus (mg)	888.6 ± 201.7	972.3 ± 427.7	0.652	0.18	1062.9 ± 253.6	1130.4 ± 394.4	0.770	0.11	857.7 ± 160.1	1177.6 ± 393.6	**0.021**	0.66	0.416	0.00
Sodium (mg)	3045.4 ± 1108.2	2801.1 ± 1017.9	0.652	0.18	2881.2 ± 1504.6	3298.1 ± 924.6	0.375	0.31	3195.6 ± 1394.7	3611.0 ± 1382.8	0.569	0.18	0.569	0.00
Potassium (mg)	2271.7 ± 511.9	2227.0 ± 950.2	0.910	0.06	2574.7 ± 703.3	2626.3 ± 1008.0	0.922	0.05	2243.3 ± 573.6	2610.0 ± 779.1	0.519	0.20	0.742	0.00
Magnesium (mg)	248.9 ± 112.9	226.6 ± 105.5	0.820	0.10	278.9 ± 130.2	269.8 ± 83.8	1.000	0.02	203.7 ± 49.8	287.4 ± 120.0	**0.042**	0.59	0.263	0.00
Iron (mg)	9.8 ± 1.9	11.6 ± 4.9	0.203	0.45	11.2 ± 5.1	13.5 ± 4.3	0.432	0.27	10.2 ± 2.4	14.1 ± 2.7	**0.005**	0.77	0.654	0.00
Zinc (mg)	6.2 ± 2.0	6.8 ± 3.0	0.734	0.14	7.4 ± 2.3	7.5 ± 3.0	1.000	0.02	5.6 ± 1.4	10.2 ± 3.7	**0.001**	0.86	**0.018**	0.01
Selenium (μg)	58.3 ± 20.8	64.2 ± 27.1	0.734	0.14	70.1 ± 17.7	75.8 ± 29.9	0.695	0.15	54.3 ± 25.4	64.6 ± 22.8	0.519	0.20	0.957	0.00
Cholesterol (mg)	211.8 ± 83.7	206.1 ± 98.6	0.910	0.06	237.2 ± 37.9	225.8 ± 128.4	0.432	0.27	194.4 ± 112.1	224.3 ± 98.0	0.850	0.07	0.792	0.00
SFA (g)	8.8 ± 3.7	10.2 ± 4.8	0.496	0.26	12.7 ± 4.4	12.2 ± 7.9	0.922	0.05	10.0 ± 4.4	13.4 ± 4.6	0.077	0.52	0.496	0.00
MUFA (g)	11.6 ± 6.8	11.8 ± 5.6	0.820	0.10	14.4 ± 7.0	11.7 ± 5.1	0.275	0.37	10.0 ± 3.5	11.6 ± 3.9	0.339	0.29	0.356	0.00
PUFA (g)	11.0 ± 5.9	12.2 ± 7.1	0.426	0.30	12.7 ± 6.7	12.7 ± 8.0	1.000	0.02	9.5 ± 4.0	13.7 ± 7.0	0.204	0.38	0.647	0.00
Omega-3 fatty acids (g)	1.9 ± 1.9	2.1 ± 1.6	0.652	0.18	1.8 ± 0.8	3.7 ± 5.8	0.625	0.18	1.2 ± 0.5	2.5 ± 2.4	0.151	0.43	0.742	0.00
Omega-6 fatty acids (g)	9.1 ± 4.1	10.0 ± 5.8	0.820	0.10	10.8 ± 6.0	9.0 ± 2.6	0.432	0.27	8.2 ± 3.7	11.0 ± 4.4	0.266	0.34	0.327	0.00

Values are presented as mean ± SD.

DiEx, nutrition and exercise education only; DiExSp, nutrition and exercise education plus dietary supplementation; SFA, Total saturated fatty acids; MUFA, Total monounsaturated fatty acids; PUFA, Total polyunsaturated fatty acids; SD, standard deviation.

[a] *p* values were obtained using the Wilcoxon signed-rank test for within-group comparisons between baseline and 12 weeks.

[b] Effect sizes (*r*) corresponding to the Wilcoxon signed-rank test were calculated as $|Z|/\sqrt{n}$; values of *r* were interpreted as small (0.10-< 0.30), medium (0.30-< 0.50), and large (≥ 0.50).

[c] *p* values were obtained using the Kruskal–Wallis test for comparison of changes from baseline to 12 weeks among the three groups.

[d] Effect sizes (ε^2) corresponding to the Kruskal–Wallis test were calculated as H / ($N^2 - 1$), where H is the test statistic and N is the total sample size; values of ε^2 were interpreted as small (0.01-< 0.06), medium (0.06-< 0.14), and large (≥ 0.14).
Boldface indicates statistical significance ($p < 0.05$).

Table S3. Changes in physical function from baseline to 12 weeks within and between groups.

**Variables**	**Control group (*n* = 9)**		***p* [a]**	***r* [b]**	**DiEx group (*n* = 10)**		***p* [a]**	***r* [b]**	**DiExSp group (*n* = 12)**		***p* [a]**	***r* [b]**	***p* [c]**	**ε^2 [d]**
	**Baseline**	**12-week**			**Baseline**	**12-week**			**Baseline**	**12-week**				
SPPB														
Total score	9.9 ± 0.9	9.7 ± 1.4	1.000	0.18	10.3 ± 1.3	11.0 ± 0.7	0.151	0.57	10.4 ± 0.5	10.6 ± 0.5	0.151	0.95	0.373	0.00
Five-time chair stand test (seconds)	8.6 ± 3.4	9.1 ± 3.6	0.641	0.20	8.3 ± 3.8	6.8 ± 1.0	0.432	0.27	6.9 ± 2.6	7.1 ± 1.8	1.000	0.00	0.804	0.00
Balance test: feet together (seconds)	10.0 ± 0.0	10.0 ± 0.0	-	-	10.0 ± 0.0	10.0 ± 0.0	-	-	10.0 ± 0.0	10.0 ± 0.0	-	-	1.000	0.00
Balance test: semi-tandem stance (seconds)	10.0 ± 0.0	10.0 ± 0.0	-	-	10.0 ± 0.0	10.0 ± 0.0	-	-	10.0 ± 0.0	10.0 ± 0.0	-	-	1.000	0.00
Balance test: tandem stance (seconds)	10.0 ± 0.0	10.0 ± 0.0	-	-	10.0 ± 0.0	10.0 ± 0.0	-	-	10.0 ± 0.0	10.0 ± 0.0	-	-	1.000	0.00
4-meter walking speed (seconds)	6.6 ± 0.9	6.5 ± 0.8	0.445	0.30	6.1 ± 0.8	5.8 ± 0.7	0.289	0.38	6.4 ± 0.9	6.0 ± 0.8	0.110	0.48	0.726	0.00

Values are presented as mean ± SD.
DiEx, nutrition and exercise education only; DiExSp, nutrition and exercise education plus dietary supplementation; SPPB, Short Physical Performance Battery; SD, standard deviation.
[a] *p* values were obtained using the Wilcoxon signed-rank test for within-group comparisons between baseline and 12 weeks.
[b] Effect sizes (*r*) corresponding to the Wilcoxon signed-rank test were calculated as $|Z|/\sqrt{n}$; values of *r* were interpreted as small (0.10-< 0.30), medium (0.30-< 0.50), and large (≥ 0.50).
[c] *p* values were obtained using the Kruskal–Wallis test for comparison of changes from baseline to 12 weeks among the three groups.
[d] Effect sizes (ε^2) corresponding to the Kruskal–Wallis test were calculated as H / ($N^2 - 1$), where H is the test statistic and N is the total sample size; values of ε^2 were interpreted as small (0.01-< 0.06), medium (0.06-< 0.14), and large (≥ 0.14).
Boldface indicates statistical significance ($p < 0.05$).
